# Effect of Paclobutrazol on Growth and Storage Root Yield of Sweet Potato (*Ipomoea batatas* (L.) Lam.) ‘Kuri Kogane’ Grown in Planting Bags

**DOI:** 10.3390/plants15182878

**Published:** 2026-09-20

**Authors:** Kiriya Sungthongwises, Chanon Lapjit, Khomsorn Lomthaisong

**Affiliations:** 1 Agronomy Section, Faculty of Agriculture, Khon Kaen University, Khon Kaen 40002, Thailand; 2 Horticulture Section, Faculty of Agriculture, Khon Kaen University, Khon Kaen 40002, Thailand; lchano@kku.ac.th; 3 Department of Biochemistry, Faculty of Science, Khon Kaen University, Khon Kaen 40002, Thailand; kholom@kku.ac.th

**Keywords:** container cultivation, economic yield, Kuri Kogane, plant growth regulator, sweet potato

## Abstract

Excessive vegetative growth in sweet potato often disrupts source–sink relationships, reducing assimilate allocation to storage roots in containerized systems. This study evaluated the effects of paclobutrazol (PBZ)—a gibberellin biosynthesis inhibitor—on the vegetative growth, physiological parameters, and storage root yield of sweet potato (*Ipomoea batatas* L.) cv. Kuri Kogane grown in planting bags. A completely randomized design (CRD) was implemented using PBZ concentrations of 0, 25, 50, 100, and 200 ppm. Applications at 100 and 200 ppm significantly suppressed leaf area and aboveground biomass at 2 months after planting (MAP). Conversely, 100 and 200 ppm PBZ enhanced photosynthetic pigments, significantly increasing SPAD chlorophyll meter readings (SCMRs), with 100 and 200 ppm yielding the highest chlorophyll a and total chlorophyll contents at 1 month after planting. Consequently, 100 and 200 ppm PBZ increased fresh tuber yields to 41,933 and 42,000 kg ha^−1^, respectively. Although the Harvest Index numerically increased to 0.61–0.63, this non-significant trend was driven primarily by the reduction in aboveground biomass rather than an increase in tuber yield. Furthermore, 200 ppm PBZ promoted foliar nutrient accumulation (N, P, K, Ca, and Mg) and elevated tuber protein content to a maximum of 6.32%, while sweetness (°Brix) and carotenoid content remained unchanged. In conclusion, PBZ application effectively restricts excess shoot growth and enhances storage root yield and protein content in container-grown sweet potato.

## 1. Introduction

Sweet potato is an economically important food crop due to its nutritional value. It is a rich source of carbohydrates, which provide energy, and is high in vitamins A, B2, and folate, with levels second only to leafy green vegetables. It also contains vitamin C, which nourishes body tissues and helps the body absorb carotenoids more effectively [1], as well as fiber, iron, and protein, and antioxidants such as carotenoids, anthocyanins, and phenolic compounds, which have properties that can help prevent non-communicable diseases (NCDs) such as diabetes, hypertension, and heart disease [2]. Kuri Kogane is a premium variety of Japanese sweet potato, valued for its chestnut-like flavor and silky, golden interior. The average total yield is 25 t ha^−1^, with a market price of roughly 30 Baht per kilogram, leading to an approximate gross income of 750,000 Baht per hectare [3]. Furthermore, sweet potato leaves are a source of many nutrients, such as protein, fiber, K, P, Ca, Mg, Fe, Mn, and Cu, and are rich in antioxidants, thus helping to reduce malnutrition [4]. They can also be used as animal feed [5]. As a result, sweet potatoes are becoming increasingly popular among consumers, both for fresh consumption and processed food products, as well as for use in the health food industry. However, sweet potato production in Thailand still faces the problem of low fresh tuber yield per rai, especially during the rainy season. Sweet potatoes often receive excessive water, causing the plants to grow more stems and leaves than normal. This results in nutrients from photosynthesis being used for stem growth instead of being accumulated in the tubers, which are the most economically valuable part of the production process. This problem significantly reduces sweet potato tuber yields [2]. In addition, traditional sweet potato production involves complex and labor-intensive field management, particularly weed control during the first 1–2 months of cultivation, a critical period in tuber formation and yield. The harvesting process also requires manual digging and affects product quality.

To break the cycle of fatigue and reduce the use of an increasingly older labor force, solutions are needed to address these problems. The use of plant growth management technology in planting bag containers is therefore a promising approach for increasing productivity and reducing nutrient loss compared to field cultivation. Paclobutrazol (PBZ) is one of the triazole derivatives [6], a group with plant growth-regulating properties, and inhibits the synthesis of sterols and gibberellins [7]. Paclobutrazol has been widely used in root crop species to decrease assimilate partitioning to stems, leaves, roots, and stolons but increase the partitioning of dry mass production to tubers [8]. It affects plant growth and development by altering the rate of photosynthesis and phytohormone levels [9]. An increase in tuber fresh weight and dry mass production after the use of paclobutrazol has been reported in several crops, such as elephant foot yam [10], potato [11], and carrot [12]. However, the effects of paclobutrazol on plant growth and yield are uncertain and depend on the plant condition, environmental conditions, and management practices [13]. Ribeiro et al. [14] reported that excess nitrogen causes sweet potato leaves to overgrow and reduces root yield, but applying paclobutrazol (PBZ) along with moderate nitrogen (50 kg ha^−1^) controls growth and boosts root production across both rainy and dry seasons. In previous experiments, PBZ reduced growth in potato stems, leaves, and roots to increase assimilate partitioning directly into tubers [15]. Boonlertnirun et al. [16] found that paclobutrazol did not affect sweet potato vine length or leaf area, but it significantly impacted tuber yield and agronomic traits. Applying 200 mg L^−1^ produced the best overall results for leaf greenness, chlorophyll fluorescence, and tuber yield. Similarly, PBZ applications (200–300 mg L^−1^) effectively controlled vine length and increased chlorophyll readings. The PBZ concentration of 200 mg L^−1^ produced the highest tuber yield and root number while maximizing uniform, commercially preferred medium-sized roots (2–5 cm) [2]. As a fungicide that triggers antioxidant defenses [17,18,19], it boosts carotenoid levels in plants such as carrots [12]. Due to the properties of paclobutrazol, it has been proposed as a means of reducing stem and leaf growth. This would result in nutrients stored within the plant being transferred to the sweet potato tubers, leading to increased tuber yield. Therefore, this experiment aimed to study the effects of different concentrations of paclobutrazol on stem and leaf growth and tuber yield of Kuri Kogane sweet potato under bagged planting conditions.

## 2. Results

### 2.1. Soil Before Planting and Growth Characteristics of Kuri Kogane Sweet Potato

**Figure 1:**
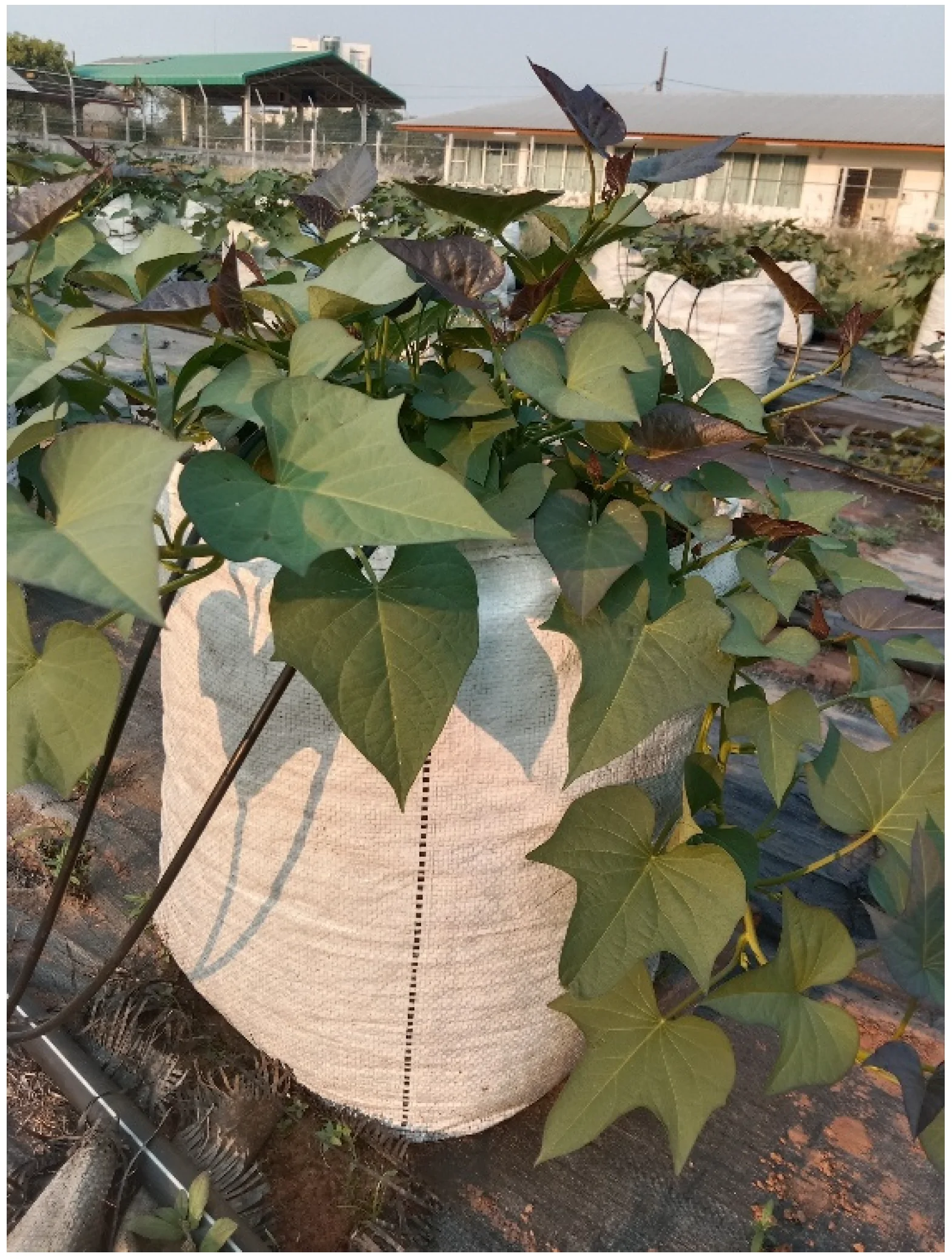
Morphology of Kuri Kogane sweet potatoes one month after planting.

The soil before planting was classified as sandy loam, with a neutral pH of 7.46. In addition, the non-saline EC was measured at 0.76 dS m^−1^. Several essential fertility nutrients, such as OM, total N, available P, and exchangeable K, were found at 11.09%, 0.47%, 0.15 mg kg^−1^, and 0.51 mg kg^−1^, respectively. However, the total concentration of heavy metals in this soil mix was within the normal range for plant growth, except that As and Hg exceeded the standard values that should be present in planting materials [20]. The effect of paclobutrazol on vegetative growth parameters at 1 month after planting Kuri Kogane sweet potato in a planting bag is shown in Figure 1. Paclobutrazol application had no significant effect on vine length, node length, the number of nodes, and leaf area per plant. SCMR values significantly increased at the 200 ppm PBZ level, peaking at 55.60, whereas 100 ppm PBZ (52.28) showed an intermediate response that did not differ significantly from lower levels and the control (49.30). At 1 MAP, control plants exhibited greater leaf area per plant (2103.40 cm^2^) than most PBZ treatments; however, response patterns varied at 2 MAP depending on the PBZ concentration (Table 1). After 2 months, significant differences were observed among PBZ treatments for vine length, SCMR, and leaf area per plant. In particular, 100 and 200 ppm concentrations of PBZ showed the highest vine lengths of 167.16 and 163.82 cm, respectively. SCMR values were significantly higher in 200 ppm PBZ-treated plants, reaching up to 51.60 compared to the control (41.04). Paclobutrazol 100 and 200 ppm applications led to a marked reduction in leaf area per plant compared to 50 ppm, to 3996.00 and 5344.10 cm^2^, respectively.

### 2.2. Chlorophyll and Carotenoid Content

Application of PBZ significantly enhanced the levels of chlorophyll a and total chlorophyll compared to the control at 1 month after planting. Paclobutrazol application showed no statistically significant effect on chlorophyll b and carotenoid content (Table 2). In addition, significant differences were recorded among the PBZ applications for chlorophyll a, chlorophyll b, and total chlorophyll contents at 2 months after planting. Paclobutrazol concentrations of 100 ppm significantly enhanced the levels of chlorophyll a but were not different from no PBZ application, 25 ppm, and 200 ppm. Meanwhile, PBZ concentrations of 25 ppm significantly enhanced the levels of chlorophyll b and were not significantly different from no PBZ application (Table 2).

### 2.3. Yield Components of Kuri Kogane Sweet Potatoes

**Figure 2:**
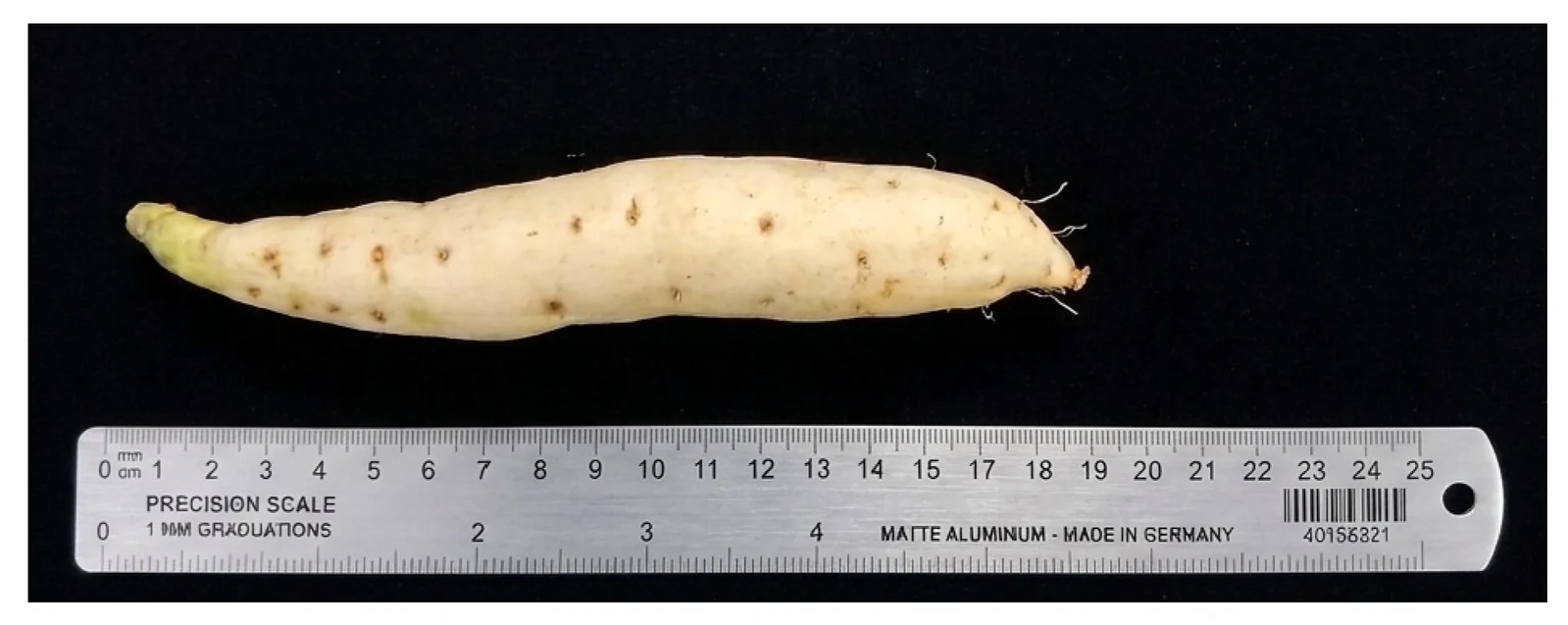
Tuber length of Kuri Kogane sweet potatoes.

Paclobutrazol concentrations of 100 and 200 ppm tended to increase the number of tubers per plant and tuber fresh weight compared with the control and lower-concentration treatments, but no significant differences were observed in the number of tubers per plant, tuber length (Figure 2), tuber diameter, tuber fresh weight, and HI (Table 3). However, the 25 and 50 ppm PBZ applications did not significantly increase the fresh and dry weights of aboveground biomass compared to the control.

### 2.4. Macronutrient and Nutritional Content

#### 2.4.1. Macronutrient Content

The use of 200 ppm paclobutrazol significantly promoted the absorption and accumulation of all major nutrients in Kuri Kogane sweet potato leaves at harvest compared with the control (0 ppm) (Table 4).

#### 2.4.2. Nutritional Content

Paclobutrazol application significantly increased protein, fat, fiber, and ash (mineral) content in Kuri Kogane sweet potatoes, while decreasing carbohydrate content (Table 5). The PBZ concentration of 200 ppm showed the highest protein content of 6.32%, while no PBZ application showed the highest carbohydrate content of 83.04%. However, all treatments showed a higher fat content of roughly 0.1–0.3 g, especially the PBZ concentration of 100 ppm. In addition, the PBZ concentration of 50 ppm showed the highest fiber and ash contents of 4.72 and 5.92%, respectively. Regarding sweetness and pigmentation, paclobutrazol had no significant effect on Brix and total carotenoid content in the sweet potatoes.

## 3. Discussion

### 3.1. Effect of Paclobutrazol on Vegetative Growth and SCMR

High variability was observed in leaf area at 2 months after planting. The pronounced variation in leaf area is primarily due to asynchronous new leaf sprouting on each plant and frequently occurs in vegetative propagation before crop canopy closure. Applications of PBZ at 100 and 200 ppm significantly reduced leaf area and aboveground biomass at 2 months after planting. However, vine length increased in PBZ-treated plants, suggesting that PBZ selectively influenced stem elongation rather than leaf expansion and biomass accumulation under these conditions. Although PBZ reduces aboveground biomass, the increase in SCMR at 100 and 200 ppm may indicate thicker leaves and a higher chlorophyll concentration per unit area [21], due to the increased development of the palisade layer in plants; the intercellular spaces decrease, resulting in more tightly packed cells and an increase in chlorophyll content per leaf area, leading to improved photosynthetic efficiency per leaf area [22]. Furthermore, 1 month after planting, PBZ application, particularly at 100 and 200 ppm, significantly increased chlorophyll A and total chlorophyll content compared to a control group. This increase in photosynthetic pigments may be attributed to PBZ slowing chlorophyll degradation and promoting chloroplast development, resulting in longer-lasting leaf greenness and photosynthetic efficiency [21]. These experimental results are consistent with the research of Boonlertnirun et al. [16], who found that the use of paclobutrazol at a concentration of 200 ppm resulted in the highest statistically significant difference in greenness (47.56%) and the highest chlorophyll fluorescence, especially chlorophyll A, when compared to the absence of paclobutrazol at 1 month after transplanting. Moreover, the experiment by Arzani and Roosta [23] found that PBZ significantly reduced leaf growth, as well as the fresh and dry weight, of mature apricot (*Prunus armeniaca* L.) compared to the control.

### 3.2. Paclobutrazol Enhances Economic Yield and Assimilate Partitioning

**Figure 3:**
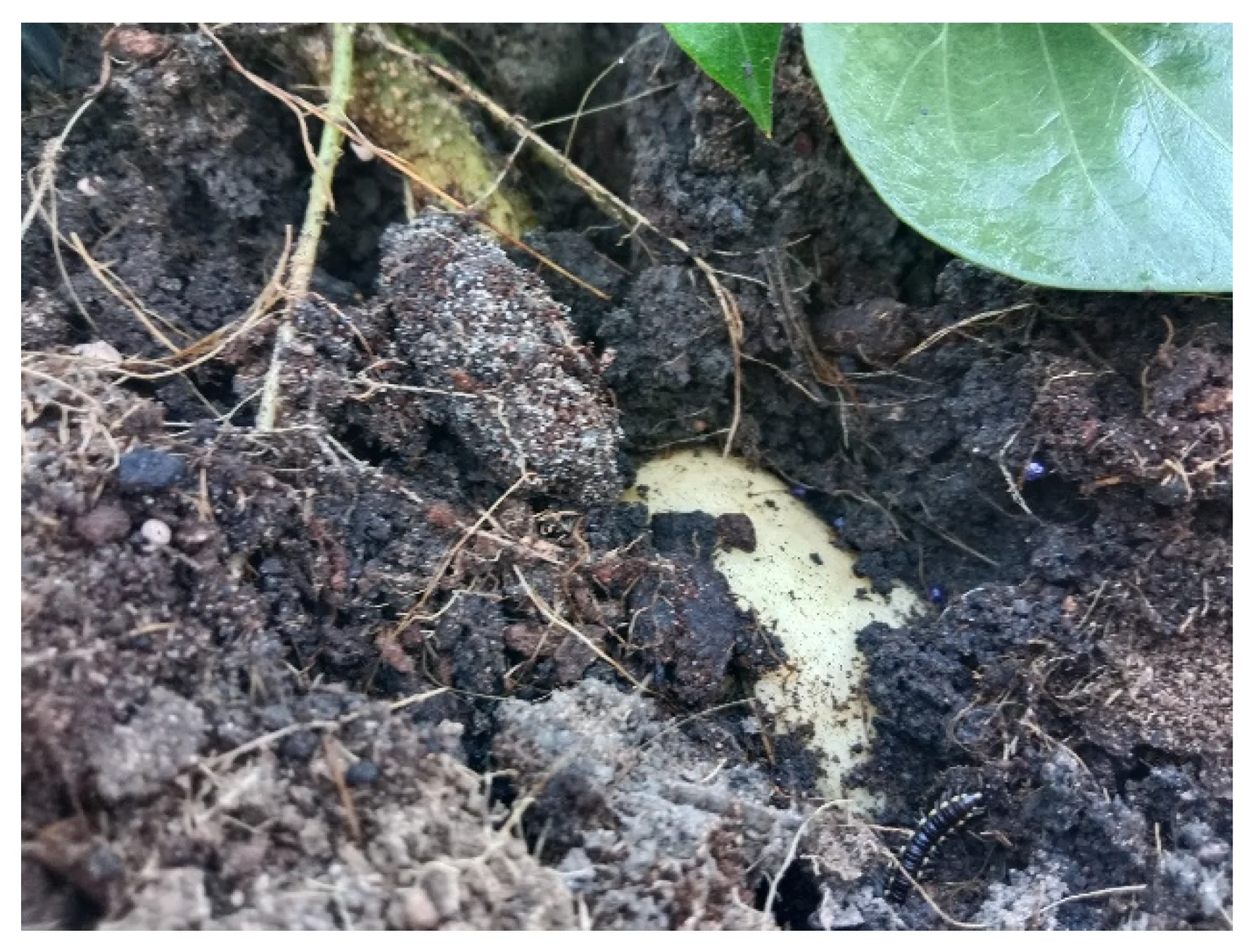
The development of Kuri Kogane superficial roots capable of forming storage.

Although differences in the number of tubers per plant, tuber size, and total fresh tuber yield were not statistically significant, the application of PBZ at 100–200 ppm induced a substantial shift in biomass allocation. Consequently, the Harvest Index (HI) exhibited a non-significant increasing trend from 0.32 in the control to 0.61–0.63 in the PBZ treatments. This upward shift in HI was primarily driven by the significant suppression of aboveground vegetative growth rather than an actual increase in tuber mass, which aligns with the non-significant effect observed for tuber yield. Fresh tuber yield exhibited a numerical upward trend, reaching approximately 41,933–42,000 kg ha^−1^ at 100–200 ppm compared to 25,200 kg ha^−1^ in the control. Rather than driving absolute yield expansion, these results suggest that PBZ primarily acts as a growth regulator that optimizes dry matter partitioning by suppressing excessive vegetative growth (source) and prioritizing assimilate transfer toward storage roots (sink). This response aligns with the established mechanism in which PBZ stimulates the development of superficial roots (>5 mm in diameter) capable of forming storage tubers [22] (Figure 3). Furthermore, PBZ enhances photosynthetic pigment concentration [24] and facilitates photoassimilate redirection [16]. In particular, 200 ppm PBZ significantly promoted leaf nutrient accumulation, most notably potassium (K), which increased to 6.82% compared to 3.60% in the control. Potassium plays an essential role in stomatal regulation, phloem loading, and starch synthesis [25]. Thus, enhanced potassium accumulation directly supports the physiological transition toward efficient sink development [26]. However, variations in tuber fresh weight reflect differences in individual sink strength and local soil moisture during development. Readers should therefore interpret these early growth trends and yield components with caution. To minimize moisture-induced variability and improve precision, future studies should increase sample replication, use uniform planting materials, or report dry tuber yield instead of fresh weight.

### 3.3. Nutritional Content of Tubers

Paclobutrazol at 200 ppm significantly increased protein content in sweet potato tubers by up to 6.32% compared to 2.04% in the control, while carbohydrate content decreased only slightly. PBZ did not significantly affect sweetness (Brix) or carotenoid content in sweet potato tubers. The increase in protein content may be due to PBZ stimulating nitrogen metabolism and nutrient uptake via the root system, thereby improving protein and mineral nutritional quality without compromising sweetness [14]. The decrease in carbohydrates may be due to increased water accumulation in tuber cells. The initial increase in size and fresh weight is usually due to cell expansion that accumulates water and minerals in the vacuoles, rather than to the synthesis and accumulation of denser starch granules, resulting in increased fresh weight while dry weight or starch content remains the same [16]. When total leaf area decreases, it affects the total photosynthetic energy (source capacity limit). Although PBZ makes leaves darker green due to denser chlorophyll, a side effect is a significant decrease in the leaf area index (LAI) and leaf size. The total starch and sugar that the plant can produce (source strength) is based on the total light-receiving area. When the total light-receiving area decreases, the total photosynthetic rate of the plant does not increase, so the sucrose nutrients transported to the tubers remain limited. Furthermore, PBZ disrupts plant hormone balance by reducing gibberellin and may affect cytokinin or auxin, even though it stimulates tuber formation. However, it does not directly promote the function of enzymes that convert sucrose into starch, such as ADP–glucose pyrophosphorylase (AGPase) or starch synthase. Therefore, the nutrients transported are in the form of sucrose or other water-absorbing substances but are not condensed into stored starch [27].

## 4. Materials and Methods

### 4.1. Experimental Site

To investigate the effects of paclobutrazol, Kuri Kogane sweet potato slips were transplanted at five concentrations, 0, 25, 50, 100, and 200 ppm, at the vegetable farm section, Department of Horticulture, Faculty of Agriculture, Khon Kaen University, Khon Kaen, Thailand. The experimental site is situated at latitude 16°25.00′ N and longitude 102°50.00′ E in 2025 and 2026. The rainy season runs from May to October, followed by the winter and dry seasons. Over the crop growth periods at the end of 2025 and at the beginning of 2026, rainfall was 101.30 mm and 154.90 mm, respectively. The maximum recorded temperatures were 34.54 and 33.50 °C, and the minimum temperatures were 24.04 and 22 °C in 2025 and 2026, respectively. To prevent weeds, the study area was covered with plastic, and a black plastic net was placed along the aisle of each plot.

### 4.2. Growing Media and Sweet Potato Slip Preparation

Planting soil was mixed with cow manure, filter cake sludge, and chopped coconut husk in a 2:1:1:1 ratio. The mixture was thoroughly mixed using a horizontal planting mix mixer Super Line IE1 Series, Mitsubishi Electric Automation (Thailand) Co., Ltd. (Bangkok, Thailand) (MEATH) then filled into 25 L planting bags (30 cm diameter × 35 cm high) for 100 bags. Planting bags containing various types of growing media, each weighing 22 kg bag^−1^, were randomly placed at a distance of 50 × 100 cm. The growing media were collected and preserved in plastic bags for analysis of their chemical properties before planting. Soil pH was measured with a pH meter; organic matter (OM) by the Walkley and Black method [28]; electrical conductivity (EC) by an EC meter; total nitrogen (N) by the Kjeldahl method [29]; available phosphorus (P) by the Bray II and molybdenum-blue method [30]; and exchangeable potassium (K) and total chromium (Cr), arsenic (As), cadmium (Cd), lead (Pb), and mercury (Hg) by wet digestion and inductively coupled plasma optical emission spectroscopy (ICP-OES).

### 4.3. Field Management

The sweet potato slips (25–30 cm) were planted at a depth of 10–15 cm in the soil mix, using one slip per bag. The trial was carried out in a completely randomized design with five treatments, each replicated 20 times, for two crops, and a drip irrigation system was installed to enhance vegetative growth after transplanting. Weed control was performed manually for 1 month after planting and then regularly every month. At 30 and 45 days after planting the sweet potatoes, the commercial NPK fertilizer (46.88 kg N ha^−1^ + 46.88 kg P ha^−1^ + 159.38 kg K ha^−1^) recommended by the Department of Agriculture, Bangkok Province, Thailand, was applied via the drill method. Paclobutrazol was applied according to the experimental design at 2, 4, and 6 weeks after transplanting, using the drenching method with 200 mL per bag per time (total 600 mL plant^−1^), in the morning. Paclobutrazol was dissolved in water, and the control group (0 ppm) received the same amount of water without PBZ.

### 4.4. Data Collection

Vine length, node length, number of nodes per plant, SPAD chlorophyll meter reading (SCMR), leaf area (LA), chlorophyll A, chlorophyll B, total chlorophyll, and carotenoids were measured at 30 and 60 days after planting. The vine length was measured from the base of the stem to the tip of the shoot. SCMRs were taken in the middle of the vine with fully grown leaves, and the average was calculated. Chlorophyll measurement was modified according to the method of Arnon [31] in 2.5. The LA was estimated using an LI-Cor 3100 leaf area meter (LI-COR Inc., Lincoln, NE, USA). At the harvest stage, several parameters were recorded: the number of tubers per plant, tuber length, tuber diameter, tuber fresh weight, fresh and dry weight of aboveground biomass, and Harvest Index (HI). Additionally, total N was determined according to Bremner and Mulvaney’s method [29], total P using wet digestion and the spectrophotometer method [32,33], and total K, Ca, and Mg using wet digestion (nitric perchloric) and an atomic absorption spectrophotometer [33]. Ash, fat, and fiber were determined using the Weende method [34]; the nutritional content of the tuber, such as protein content, was determined via combustion (AOAC official method 990.03), and carbohydrates were determined via calculation by subtracting the sum of the percentage moisture, crude protein, ether extract, crude fiber, and ash content [35]. The sweetness value was measured using a hand refractometer [35] in percent Brix. The extraction and isolation of total carotenoids in sweet potato tubers was carried out following the method of Rodriguez-Amaya and Mihoko and Saini and Keum [36,37].

### 4.5. Pigment Analysis

The sample leaves of the sweet potatoes growing in field conditions were taken from leaf position numbers 3–4. The collected leaves were cut into small 100 mg pieces, leaving the mid-ribs, and ground with 10 mL of 80% acetone (*v*/*v*). The sample solution was filtered through Whatman No. 1 filter paper, and the final volume was collected. The absorption of the carotenoids, chlorophyll a, and chlorophyll b was read at 440, 645, and 663 nm with a UV-Vis spectrophotometer (Model i3, Hanon, Jinan, China) using 80% acetone as a blank. The following formulas were used to calculate total chlorophyll content, chlorophyll a, chlorophyll b, and carotenoids [38], with all the photosynthetic pigment contents expressed in mg gFW^−1^:

Chlorophyll a (mg gFW^−1^) = [(12.7 × OD663) − (2.69 × OD645)] × [V/(1000 × W)] (1)

Chlorophyll b (mg gFW^−1^) = [(22.9 × OD645) − (4.68 × OD663)] × [V/(1000 × W)] (2)

Total chlorophyll (mg gFW^−1^) = [(20.2 × OD645) + (8.02 × OD663)] × [V/(1000 × W)] (3)

Carotenoid (mg gFW^−1^) = (4.69 × OD440) − [0.268 × [(20.2 × OD645) + (8.02 × OD663)]] × [V/(1000 × W)] (4)

where

OD440 = absorbance at 440 nm;

OD645 = absorbance at 645 nm;

OD663 = absorbance at 663 nm;

V = final volume of 80% acetone in chlorophyll extract;

W = fresh weight of leaf materials used (mg).

### 4.6. Measuring Carotenoid Content in Fresh Tuber Products

The extraction and separation of carotenoids in sweet potato using diethyl ether was performed by grinding 0.2 g of fresh sweet potato tubers in 2 mL of ethanol. The ground sample was then poured into a test tube. Approximately 2 mL of diethyl ether was added to one test tube, and the tube was shaken. Afterward, 3–4 mL of 2% NaCl was added, and the tube was shaken for approximately 5–7 min; layer separation occurred. The top layer (diethyl ether layer) was transferred to another test tube, and 4–5 mL of distilled water was added. After waiting approximately 2–3 min, the ethanol present in the diethyl ether layer was separated. This resulted in a two-layered solution: the top layer being the diethyl ether layer and the bottom layer being the aqueous layer containing ethanol. Next, we aspirated the top layer (diethyl ether layer), which was yellow, orange, or red in color, and stored it in the dark at −20 °C until measurement of the extracted carotenoid.

To measure the extracted carotenoid, we evaporated the carotenoid solution dissolved in diethyl ether using nitrogen gas (N_2_). We dissolved the carotenoid in diethyl ether to a total volume of 3 mL. Then, we measured the absorbance using a spectrophotometer. At a wavelength of 450 nm, the concentration and total carotenoid content per unit weight of the sample were calculated using the methods of Rodriguez-Amaya and Mihoko and Saini and Keum [36,37] as follows:

C = A/εl(5)

Total carotenoids = C × V(6)

Carotenoid per wet weight = Total carotenoid/Wet weight(7)

Here,

A = absorbance value at a wavelength of 450 nm.

ε = 0.25 Specific Extinction Coefficient.

l = distance the light passes through the solution; 1 cm.

C = concentration of substance in solvent (µg/mL).

V = volume of solution obtained from extraction; 3 mL.

Total carotenoids = total carotenoid content in solution (µg).

Carotenoid per wet weight = carotenoid content per 1 g of wet weight of sweet potato (µg/g).

### 4.7. Statistical Analysis

The data were analyzed via one-way Analysis of Variance (ANOVA) using the Statistix 10 software. Comparative analysis of the results was carried out using Tukey’s honestly significant difference (HSD) test at the 5% level of significance; that is, a *p*-value ≤ 0.05 was considered statistically significant.

## 5. Conclusions

Applying paclobutrazol (PBZ) at 100 and 200 ppm significantly reduced leaf area per plant and both fresh and dry aboveground biomass at 2 months after planting (MAP). Concurrently, PBZ altered biomass partitioning by doubling the Harvest Index (HI) from 0.32 in the control to 0.61–0.63 at 100–200 ppm, despite non-significant differences in total fresh tuber yield, tuber number, and tuber size. Photosynthetic capacity was enhanced at 1 MAP under PBZ treatments, as evidenced by significantly higher SPAD chlorophyll meter readings (SCMRs) at 100 and 200 ppm and peak chlorophyll a and total chlorophyll levels. Furthermore, 200 ppm PBZ promoted maximum foliar accumulation of major macronutrients (N, P, K, Ca, and Mg), with potassium (K) reaching 6.82%, which was associated with an increase in tuber protein content to 6.32% (compared to 2.04% in the control). Conversely, 50 ppm PBZ maximized tuber fiber (4.72%) and ash (5.92%) contents, while total soluble solids (Brix) and total carotenoid levels remained unaffected across treatments. Overall, PBZ functions primarily as a canopy management tool that optimizes source-to-sink assimilate allocation rather than expanding absolute tuber yield. A concentration of 100 ppm PBZ is recommended for practical application, as it achieves an equivalent shift in the Harvest Index (HI) and physiological benefits as 200 ppm while reducing chemical usage, input costs, and potential environmental residues.

## Figures and Tables

**Table 1:** Growth of Kuri Kogane sweet potatoes one and two months after planting.

Paclobutrazol (ppm)	1 Month					2 Months				
	Vine Length (cm)	Node Length (cm)	No. of Nodes Plant^−1^	SCMR	Leaf Area Plant^−1^ (cm^3^)	Vine Length (cm)	Node Length (cm)	No. of Nodes Plant^−1^	SCMR	Leaf Area Plant^−1^ (cm^3^)
0 (Control)	46.96 ± 22.10	2.98 ± 0.59	48.60 ± 13.43	49.30 ± 5.44 ^b^	2103.40 ± 490.25	122.60 ± 27.67 ^ab^	5.80 ± 2.36	161.60 ± 57.82	41.04 ± 5.26 ^b^	13,590.00 ± 5764.02 ^bc^
25	39.98 ± 25.59	3.06 ± 1.74	38.80 ± 19.11	48.84 ± 1.62 ^b^	1361.30 ± 956.32	129.30 ± 29.05 ^ab^	4.50 ± 2.06	156.00 ± 54.90	44.88 ± 4.51 ^ab^	43,622.00 ± 26,231.86 ^ab^
50	35.56 ± 10.86	3.10 ± 1.39	43.00 ± 16.12	49.62 ± 3.44 ^b^	1485.90 ± 319.11	115.70 ± 34.88 ^b^	4.42 ± 0.64	160.60 ± 63.65	47.04 ± 3.31 ^ab^	52,299.00 ± 28,415.47 ^a^
100	62.18 ± 5.69	3.85 ± 0.35	35.94 ± 1.66	52.28 ± 1.71 ^ab^	1584.00 ± 40.91	167.16 ± 0.61 ^a^	6.55 ± 0.42	94.46 ± 23.71	50.46 ± 1.19 ^a^	3996.00 ± 591.08 ^c^
200	56.88 ± 6.55	3.46 ± 0.40	38.74 ± 6.38	55.60 ± 0.87 ^a^	1175.40 ± 231.25	163.82 ± 11.97 ^a^	5.60 ± 0.24	98.80 ± 15.76	51.60 ± 2.09 ^a^	5344.10 ± 750.11 ^c^
F-test	ns	ns	ns	*	ns	**	ns	ns	**	**
CV (%)	33.84	32.08	31.77	6.05	33.22	17.44	26.92	35.27	7.65	73.58

SCMR = SPAD chlorophyll meter reading; CV = coefficient of variation; ns = not significant; * = significant difference at *p* < 0.05; ** = significant difference at *p* < 0.01. Different letters within the same column represent significant differences based on Tukey’s honestly significant difference (HSD) test at *p* < 0.05.

**Table 2:** Chlorophyll and carotenoid contents in the leaves of Kuri Kogane sweet potatoes after planting.

Paclobutrazol (ppm)	1 Month				2 Months			
	Chl a (mg gFW^−1^)	Chl b (mg gFW^−1^)	Tc (mg gFW^−1^)	Car (mg gFW^−1^)	Chl a (mg gFW^−1^)	Chl b (mg gFW^−1^)	Tc (mg gFW^−1^)	Car (mg gFW^−1^)
0 (Control)	0.227 ± 0.08 ^c^	0.119 ± 0.03	0.346 ± 0.11 ^c^	0.160 ± 0.01	0.781 ± 0.05 ^ab^	0.508 ± 0.05 ^ab^	1.288 ± 0.1 ^a^	0.281 ± 0.02
25	0.339 ± 0.18 ^bc^	0.134 ± 0.04	0.473 ± 0.21 ^bc^	0.158 ± 0.03	0.663 ± 0.03 ^ab^	0.563 ± 0.03 ^a^	1.225 ± 0.01 ^ab^	0.239 ± 0.03
50	0.254 ± 0.05 ^c^	0.119 ± 0.02	0.372 ± 0.07 ^c^	0.173 ± 0.01	0.529 ± 0.12 ^b^	0.434 ± 0.07 ^b^	0.963 ± 0.19 ^c^	0.211 ± 0.04
100	0.617 ± 0.06 ^ab^	0.206 ± 0.02	0.822 ± 0.09 ^ab^	0.232 ± 0.04	0.878 ± 0.15 ^a^	0.256 ± 0.02 ^c^	1.134 ± 0.17 ^b^	0.248 ± 0.03
200	0.744 ± 0.39 ^a^	0.182 ± 0.12	0.926 ± 0.27 ^a^	0.217 ± 0.05	0.755 ± 0.07 ^ab^	0.237 ± 0.04 ^c^	0.993 ± 0.11 ^bc^	0.242 ± 0.09
F-test	*	ns	**	ns	*	**	*	ns
CV (%)	44.94	38.67	28.43	17.69	13.51	10.96	11.57	19.79

CV = coefficient of variation; ns = not significant; * = significant difference at *p* < 0.05; ** = significant difference at *p* < 0.01. Different letters within the same column represent significant differences based on Tukey’s honestly significant difference (HSD) test at *p* < 0.05.

**Table 3:** Yield components and economic yield of Kuri Kogane sweet potatoes.

Paclobutrazol (ppm)	No. of Tubers Plant^−1^	Tuber Length (cm)	Tuber Diameter (cm)	Tuber Fresh Weight (kg ha^−1^)	Fresh Weight of Aboveground Biomass (kg ha^−1^)	Dry Weight of Aboveground Biomass (kg ha^−1^)	HI
0 (Control)	6.33 ± 5.86	21.93 ± 1.80	4.02 ± 0.88	25,200 ± 20,453.85	71,600 ± 27,158.06 ^a^	9440 ± 2977.97 ^ab^	0.32 ± 0.14
25	6.00 ± 4.36	27.17 ± 14.91	4.78 ± 2.16	24,800 ± 16,000.00	63,933 ± 22,543.59 ^ab^	10,270 ± 3068.99 ^a^	0.39 ± 0.22
50	5.67 ± 4.04	24.99 ± 16.85	4.86 ± 2.75	20,733 ± 18,810.99	44,267 ± 8672.56 ^ab^	8721 ± 1988.79 ^ab^	0.43 ± 0.33
100	10.00 ± 4.58	22.65 ± 2.56	4.87 ± 1.17	41,933 ± 2640.71	24,533 ± 2042.87 ^b^	4425 ± 718.39 ^b^	0.63 ± 0.01
200	9.33 ± 3.21	22.25 ± 2.28	4.72 ± 0.50	42,000 ± 3815.76	26,600 ± 1587.45 ^b^	4238 ± 278.15 ^b^	0.61 ± 0.02
F-test	ns	ns	ns	ns	*	*	ns
CV (%)	60.19	42.89	36.73	46.84	35.28	28.81	39.44

Yield (kg ha^−1^) was extrapolated from single-plant replication data (one bag = one plant) using an assumed field planting density of 20,000 plants ha^−1^ (50 × 100 cm spacing). CV = coefficient of variation; ns = not significant; * = significant difference at *p* < 0.05. Different letters within the same column represent significant differences based on Tukey’s honestly significant difference (HSD) test at *p* < 0.05.

**Table 4:** Macronutrient content in the leaves of Kuri Kogane sweet potatoes at the harvest stage.

Paclobutrazol (ppm)	N (%)	P (%)	K (%)	Ca (%)	Mg (%)
0 (Control)	2.40 ± 0.01 ^b^	0.20 ± 0.002 ^c^	3.60 ± 0.003 ^c^	1.66 ± 0.01 ^c^	0.52 ± 0.003 ^c^
25	2.41 ± 0.05 ^b^	0.25 ± 0.004 ^c^	3.68 ± 0.12 ^c^	2.98 ± 0.01 ^b^	0.79 ± 0.005 ^b^
50	2.70 ± 0.02 ^a^	0.29 ± 0.002 ^b^	3.70 ± 0.10 ^c^	2.90 ± 0.01 ^b^	0.80 ± 0.01 ^b^
100	2.41 ± 0.05 ^b^	0.25 ± 0.004 ^c^	6.27 ± 0.12 ^b^	2.95 ± 0.01 ^b^	0.78 ± 0.005 ^b^
200	2.76 ± 0.02 ^a^	0.42 ± 0.002 ^a^	6.82 ± 0.10 ^a^	3.01 ± 0.01 ^a^	0.89 ± 0.01 ^a^
F-test	**	**	**	**	**
CV (%)	1.39	1.48	1.85	0.28	0.71

CV = coefficient of variation; ** indicates a significant difference at *p* < 0.01. Different letters within the same column represent significant differences based on Tukey’s honestly significant difference (HSD) test at *p* < 0.05.

**Table 5:** Nutritional content of the tubers of Kuri Kogane sweet potatoes.

Paclobutrazol (ppm)	Carbohydrate (%)	Protein (%)	Fat (%)	Fiber (%)	Ash (%)	Brix (%)	Total Carotenoid (µg g^−1^)
0 (Control)	83.04 ± 0.14 ^a^	2.04 ± 0.03 ^e^	1.03 ± 0.01 ^c^	4.02 ± 0.11 ^b^	4.19 ± 0.05 ^c^	8.60 ± 0.20	3.17 ± 0.51
25	81.95 ± 0.04 ^b^	2.56 ± 0.02 ^d^	0.63 ± 0.01 ^d^	4.15 ± 0.04 ^b^	5.77 ± 0.09 ^a^	8.67 ± 0.42	2.72 ± 1.34
50	81.06 ± 0.11 ^d^	2.86 ± 0.03 ^c^	0.42 ± 0.01 ^e^	4.72 ± 0.04 ^a^	5.92 ± 0.09 ^a^	8.40 ± 0.20	4.28 ± 0.33
100	81.65 ± 0.04 ^c^	4.87 ± 0.03 ^b^	1.13 ± 0.01 ^a^	2.79 ± 0.01 ^d^	3.85 ± 0.02 ^d^	8.67 ± 0.42	2.72 ± 1.34
200	78.60 ± 0.02 ^e^	6.32 ± 0.05 ^a^	1.06 ± 0.01 ^b^	3.13 ± 0.01 ^c^	4.83 ± 0.02 ^b^	8.40 ± 0.20	4.28 ± 0.33
F-test	**	**	**	**	**	ns	ns
CV (%)	0.11	0.85	1.25	1.42	1.27	3.57	26.32

CV = coefficient of variation; ns = not significant; ** = significant difference at *p* < 0.01. Different letters within the same column represent significant differences based on Tukey’s honestly significant difference (HSD) test at *p* < 0.05.

## Data Availability

The original contributions presented in this study are included in the article. Further inquiries can be directed to the corresponding author.
